# Supplementary material for: Diversity and dissemination of viruses in pathogenic protozoa
Source: Nat Commun. 2023 Dec 15;14:8343. doi: 10.1038/s41467-023-44085-2 (PMC10724245; doi:10.1038/s41467-023-44085-2)
Supplement: Supplementary file 1 — Supplementary Information [file 41467_2023_44085_MOESM1_ESM.pdf]

Supplementary Information for

Diversity and dissemination of viruses in pathogenic protozoa

Senne Heeren, Ilse Maes, Mandy Sanders, Lon-Fye Lye, Vanessa Adaui, Jorge Arevalo, Alejandro Llanos-Cuentas, Lineth Garcia, Philippe Lemey, Stephen M Beverley, James A Cotton, Jean-Claude Dujardin, Frederik Van den Broeck

Frederik Van den Broeck: fvandenbroeck@gmail.com, 0032 3 247 67 94
Jean-Claude Dujardin: jcdujardin@itg.be, 0032 3 247 63 58

SUPPLEMENTARY METHODS

Landscape genomics of *Leishmania braziliensis*

To investigate the spatio-environmental impact on genetic variation among the three ancestral *L. braziliensis* populations, we extracted the 19 bioclimatic variables of the WorldClim2 database [1]. All 19 variables were extracted per locality from ~1 km spatial resolution raster maps after all layers were transformed to the same extent, resolution, and coordinate reference system (WGS 1984). Geographic distances among sampling points were calculated as great-circle distances using the geodist R-package (measure = 'haversine' [2]).

The impact of geographic distance on the genetic differentiation was assessed through Mantel tests between i) the interdeme geographic distance and the Weir-Cockerham's F_{ST}[3]; ii) the geographic distance and Bray-Curtis genetic dissimilarity among individuals. The environmental and geographic influence on the ancestral genomic population structure of *L. braziliensis* was disentangled through redundancy analysis (RDA) and generalized dissimilarity modeling (GDM) including geographic distance and a selection of bioclimatic variables to reduce model overfitting and multicollinearity. Variable selection was performed adopting two approaches: i) mod-A: an RDA-based forward selection procedure using 'ordiR2step' function of the vegan R-package [4,5]; ii) mod-M: a manual variable selection procedure, selecting variables if their added contribution increased the adjusted R-squared and if both the overall RDA model and individual variable were significant (p-val < 0.05).

Variation partitioning through RDA analysis was performed on Hellinger transformed SNP data, longitude, latitude and standardized environmental variables, using the 'decostand' and 'rda' functions from the 'vegan' R-package[4], to disentangle the influence of geography (isolation-by-distance) and climate (Isolation-by-environment). Finally, the different variance components were compared based on their adjusted R-squared and each explanatory component was tested for significance using the vegan::anova.cca function [4]. In addition to RDA variation partitioning, we constructed generalized dissimilarity models (GDMs) using the gdm R-package[6], to investigate spatio-environmental patterns of the genetic variability in a non-linear way [6,7]. The GDMs constituted a genetic distance matrix (Bray-Curtis dissimilarity of Hellinger transformed SNP genotypes) as response variable and the bioclimatic variables (as selected by the mod-A and mod-M variable selection procedures) as explanatory variables. We accounted for geographic distance effects on the genomic variability by fitting two GDMs per variable selection approach including and excluding the inter-individual geographic distance matrix. Relative variable importance in each GDM was estimated based on the I-spline basis function (i.e., the maximum height of the response curves) along with uncertainty assessment by performing 1000 iterations of each GDM model [8].

Based on the most important environmental variables influencing the population genomic structure (variables selected in mod-A and mod-M), we attempted to estimate and project patches of suitable habitat for *L. braziliensis* within our study region based on bioclimatic data from the present[1], the Last Glacial Maximum (LGM) [9,10] and Last Interglacial

(LIG) [10,11] periods. Ecological niche models (ENMs) were constructed using Maxent v.3.4.3, as implemented in the dismo R-package [12,13]. The following parameters were set: 'linear', 'quadratic', 'product', 'threshold', 'hinge', 'responsecurves', 'jackknife', 'replicatetype'=crossvalidate, 'replicates'=10, 'writebackgroundpredictions'=true, 'betamultiplier'= 1, 1.5 and 2 (i.e regularization multiplier, rm). The models were generated based on presence (coordinates of sample localities) and pseudo-absence data (10,000 randomly sampled background points). The habitat suitability projections were constructed by averaging the predictions from all 10 replicates on the present-day, LGM and LIG environmental data. Finally, we accounted for the uncertainty of sampling localities by adding some noise to isolates with non-unique coordinates. This was done by adding a random jitter (factor = 0.01) to all duplicated sampling localities.

SUPPLEMENTARY RESULTS

Natural genome diversity of *L. braziliensis*

Sequence reads were aligned against the *L. braziliensis* M2904 reference genome [14], resulting in a median coverage of 58x (min=35x, max=121x). Variant discovery was done with GATK HaplotypeCaller to uncover high-quality Single Nucleotide Polymorphisms (SNPs) and small insertions/deletions (INDELs). The number of SNPs identified between each genome and the M2904 reference genome was relatively consistent across the panel, varying between 97,777 SNPs in CEN002 and 109,798 SNPs in LC2319 (median = 105,096; mean = 106,530). Exceptions were isolates PER231 (126,178), LC2318 (128,544) and CUM68 (134,882) that showed larger SNP densities and double the number of heterozygous sites compared to the other isolates (Supp. Fig. 14A,B). When investigating the genome-wide distribution of normalized allele frequencies at heterozygous sites (which should be centered around 0.5 in diploid individuals) [15], we discovered that isolates CUM68, LC2318 and PER231 showed allele frequencies that deviated from this diploid expectation. Isolates CUM68 and LC2318 showed allele frequency distributions with modes around 0.25, 0.5 and 0.75 that are symptomatic of tetraploidy, while isolate PER231 showed a skewed allele frequency distribution (Supp. Fig. 15A-D). We could not unequivocally rule out if these patterns were the result of contamination or a mixed infection. Removing these three isolates for downstream analyses, the resulting dataset comprised a total of 407,070 SNPs and 69,604 bi-allelic INDELs called across the remaining 76 genomes. Respectively 41.5% and 5.7% of the SNPs and INDELs were found in the coding region of the genome, including 437 SNPs and 55 INDELs with a deleterious impact (e.g. introducing stop codons). Most of these deleterious mutations were rare in our panel of parasites (83.6% being at <= 1% MAF), and the remaining 80 mutations were not linked to either parasite population structure or LRV infection status (Supp. Data 15).

__The role of geography and environment in partitioning *L. braziliensis* diversity__

Upon the strong signatures of geographical isolation of the three ancestral components of *L. braziliensis* (Fig 4A) and the lack of association between the inter-population (great-circle) geographic distance and the Weir & Cockerham's F_{ST} (Supp Fig. 16; Supp Data 16), we investigated the differential influence of geography and environmental variables on the parasite population structure in the region. When addressing the inter-individual association of geographic distance with genetic distance (Bray-Curtis dissimilarity of SNP genotypes) we picked up a pattern resembling case-IV isolation-by-distance (i.e., increasing genetic distance with geographic distance up to a certain point after which the relationship weakens down; Supp. Fig. 17; Supp. Data 17). This revealed that isolation-by-distance mainly plays a role within populations over distances up to ca. 500km, while IBD diminishes on an inter-population level when geographic distances become too great (> 500km).

To investigate what other factors besides geography influenced the population divergence among the ancestral populations, we investigated the potential impact of the abiotic environment through RDA-based variation partitioning (main text) and GDM analysis (genetic dissimilarity modelling), including geographic distance and 19 bioclimatic variables (Supp. Data 5). Two variable selection approaches were adopted to reduce model overfitting and multicollinearity among bioclimatic variables (Supp. methods). The mod-A variable selection initially resulted in six variables, although they revealed large variance inflation factors (vif) which prompted us to remove variables with a vif > 10 in a stepwise manner, retaining only two variables: 'isothermality' (bio3) and 'Precipitation of the driest month' (bio14). In contrast, the mod-M approach resulted in a final selection of five variables, each with vif < 10: 'isothermality' (bio3), 'Precipitation of the driest month' (bio14), 'precipitation of warmest quarter' (bio18), 'precipitation seasonality' (bio15), 'Annual mean diurnal range' (bio2) (Supp. Data 6).

Genetic dissimilarity modeling revealed overall similar patterns in explaining the genomic variability by the environment relative to geography as described by the RDA models. The GDMs based on the mod-A selected variables (bio3 and bio14) were able to explain 55.86% (excluding geography) to 65.38% (including geography) of the genomic deviance (null deviance), meaning the combined environmental component explains more of the genomic variability than geography. Nevertheless, the height of the variable response curves (i.e. the measure of variable importance) indicate that geography explains more of the genomic deviance than most environmental variables separately (Supp. Fig 18). In parallel, the GDMs for the mod-M selected variables (bio3, bio14, bio2, bio15 and bio18) revealed similar results explaining 59.98% (excluding geography) to 65.04% (including geography) of the genomic deviance.

To complete the analysis on the spatio-environmental structuring of the ancestral populations we constructed three ENMs based on partially jittered isolate coordinates and four present-day bioclimatic variables (bio3, bio14, bio2 and bio18) (Supp Data 18; Supp.

Methods), differing in their regularization multiplier parameter (rm; set to 1, 1.5 and 2). All three cross validated models showed high AUC values (area under the receiver operating curve; Supp. Data 19; Suppl Fig. 19), an indication of strong model performance. The variable contribution and permutation importance along with a jackknife test revealed that bio18 and bio14 were consistently the two variables contributing most in the suitability prediction of the maternal parasite populations (Supp. Data 18; Supp. Figure 19). In accordance with the different variation partitioning models (RDA and GDM), revealing the key variables in explaining the population divergence, our model projections of habitat suitability on present-day bioclimatic data showed that regions of suitable (abiotic) habitat coincided with tropical rainforests, as predicted by the Köppen-Geiger climate classification, where the three ancestral populations were surrounded by less suitable tropical monsoon and savannah forests (Fig. 4). Moreover, the suitability predictions on LGM and LIG bioclimatic data revealed a strong cyclical alternation of suitable habitat with a wider distribution during climatological warmer time periods (LIG, present) and strong habitat fragmentation during colder periods (LGM) (Supp. Fig 9). Such alternation in habitat extent could, in accordance with the ecological divergence between *L. peruviana* and *L. braziliensis* [14], have played a part in the further divergence of *L. braziliensis* into these distinct populations.

Quality assessment of the *Leishmania* virus 1 (LRV1) genome assemblies

The LRV1 genomes included in this study were generated through dsRNA extraction from 31 LRV1-positive *L. braziliensis* isolates that were re-cultured. Following total RNA sequencing, we performed a *de novo* assembly and LRV1 contig extraction by a local BLAST search (see Methods). The assembly quality of the genomes was examined by investigating the coverage of mapped (paired) reads, SNP counts after mapping, comparison with analogous genomic regions to ~1kb sequences obtained through conventional Sanger sequencing and by read-based computational assembly improvement using Pilon [16].

From the average 0.04% of reads that mapped against their respective LRV1 contig (Supp. Data 9), an average of 85.7% (81.3% - 91.5%) of the reads were properly paired (i.e., correctly oriented reads with respect to each other and with proper insert sizes). In addition, most LRV1 strains contained few SNPs (zero to three) of which all were heterozygous except one. PER130 showed one homozygous SNP, which was located at the 5' end (position 6) of the assembled genome which was trimmed in downstream analyses. However, in LRV-Lb-LC2321 we encountered 51 heterozygous SNPs (data not shown), suggesting the possibility of a mixed viral infection (i.e. two LRV1 strains present in one parasite isolate of which one is much lower in abundance than the other). To examine this in more detail, we extracted the reads mapping to the potential chimeric LRV1 genome and re-assembled the reads using a recently developed strain-resolving *de novo* assembler [17], developed to extract various viral strains from mixed infection samples. This resulted in two LRV1 contigs: LC2321.1 (5,260bp) and LC2321.2 (4,738bp), and considerably dropped the number of heterozygous SNPs found in both strains. LC2321.1 showed nine heterozygous SNP of which eight were located on a

non-resolved part of the LC2321.2 genome. LC2321.2 on the other hand did not reveal any SNPs. Furthermore, the remaining quality statistics of both strain resolved genomes from LC2321 were similar to the other assemblies (Supp. Data 9).

The sequence identity of the assembled genomes was assessed by comparison with analogous ~1kb (1197bp) sequences obtained through conventional Sanger Sequencing. This revealed for 83.8% (26/31) of the genomes a sequence identity of 100% (Supp. Data 9). The remaining five genomes encompassed both genomes of CUM65 and LC2321 (mixed LRV1 infections) and PER212. For CUM65, we observed a sequence identity of 99% (12 mismatches) and 86% (152 mismatches) for LRV1-Lb-CUM65.1 and LRV1-Lb-CUM65.2, respectively. For LC2321, both resolved strains showed a sequence identity of 99% with three and ten nucleotide mismatches in LRV1-Lb-LC2321.1 and LRV1-Lb-LC2321.2, respectively. These mismatches were unique to each strain, which might indicate the Sanger sequence is a chimera of both strains. Finally, the 99% identity of PER212 with its respective partial Sanger sequence showed only one nucleotide mismatch, not corresponding to the identified heterozygous SNP, suggesting a badly called base during the Sanger sequencing.

SUPPLEMENTARY FIGURES

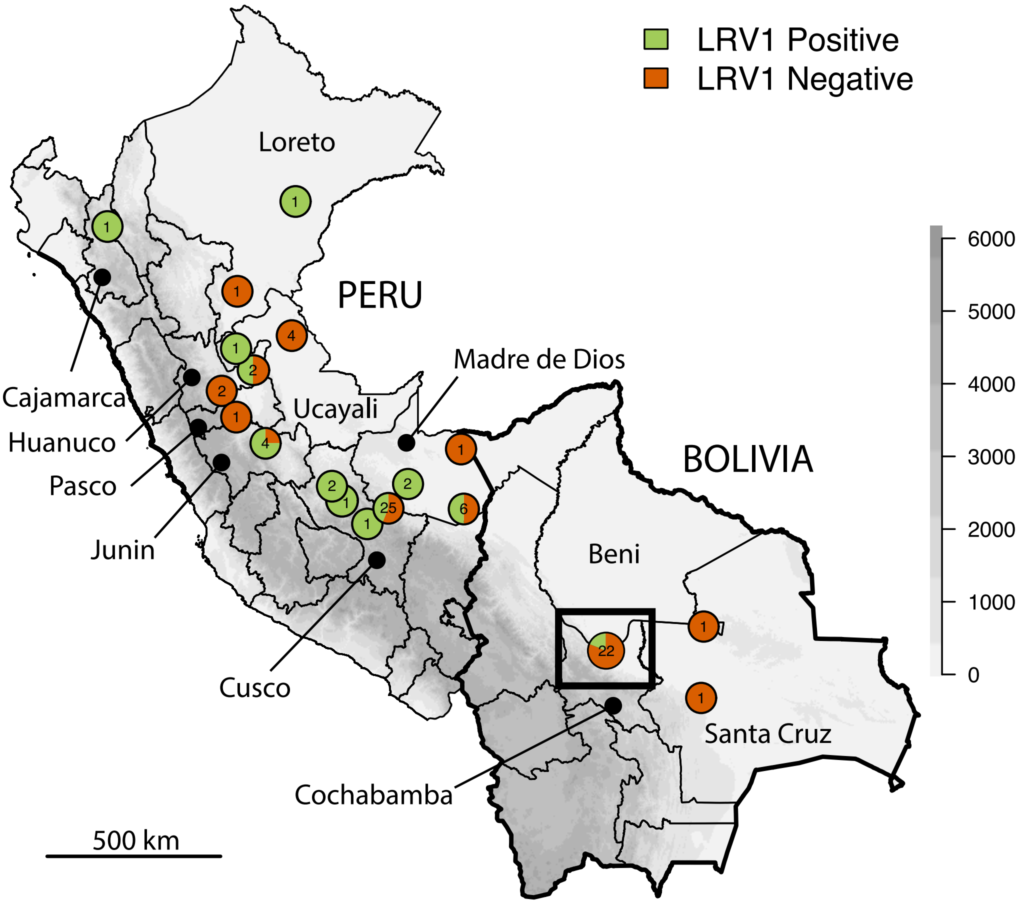

Supplementary Figure 1. Geographic origin of 79 *L. braziliensis* isolates from Peru and Bolivia that were included in this study. Rectangular box indicates the location of the Isiboro National Park that extends between the Departments of Cochabamba and Beni. Gray-scale represents altitude in meters. Country-level data for Peru and Bolivia, including administrative boundaries and altitude, were available from: http://www.diva-gis.org/Data.

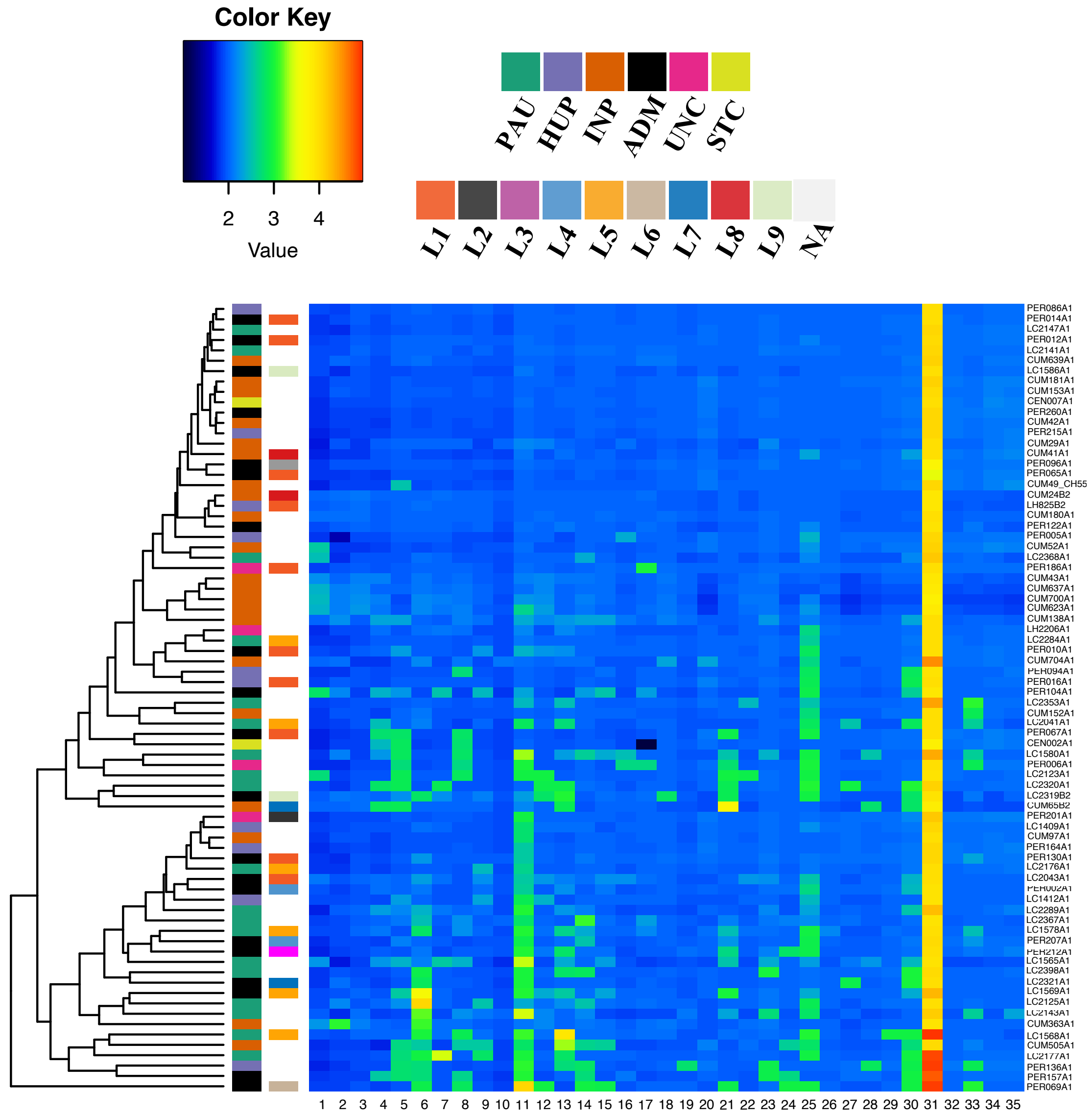

Supplementary Figure 2. Variation in chromosome copy numbers in a panel of 76 *L. braziliensis* genomes. Isolates were clustered according to similarity in somy estimation with aneuploid individuals at the bottom of the heatmap and overall diploid individuals at the top of the heatmap. Chromosome 31 was highly polysomic with at least four copies present in all isolates, a consistent observation in *Leishmania* [18,19]. Chromosomes 10, 26, 32 and 34 were disomic in all isolates. Excluding chromosome 31, we found 21 isolates that were diploid and 25 isolates with at least 5 polysomic chromosomes, including 6 isolates with at least 10 polysomic chromosomes. Coloured boxes on the left of the heatmap represent: *Left* - the inferred parasite groups PAU (green), INP (orange), HUP (purple), STC (yellow-green), ADM (black) and UNC (pink); *Right* - the identified LRV1 lineages encountered in each *L. braziliensis* isolate. I (orange), II (dark gray), III (pink), IV (steelblue), V (yellow), VI (beige), VII (dark blue), VIII (red), IX (light green), NA (light gray).

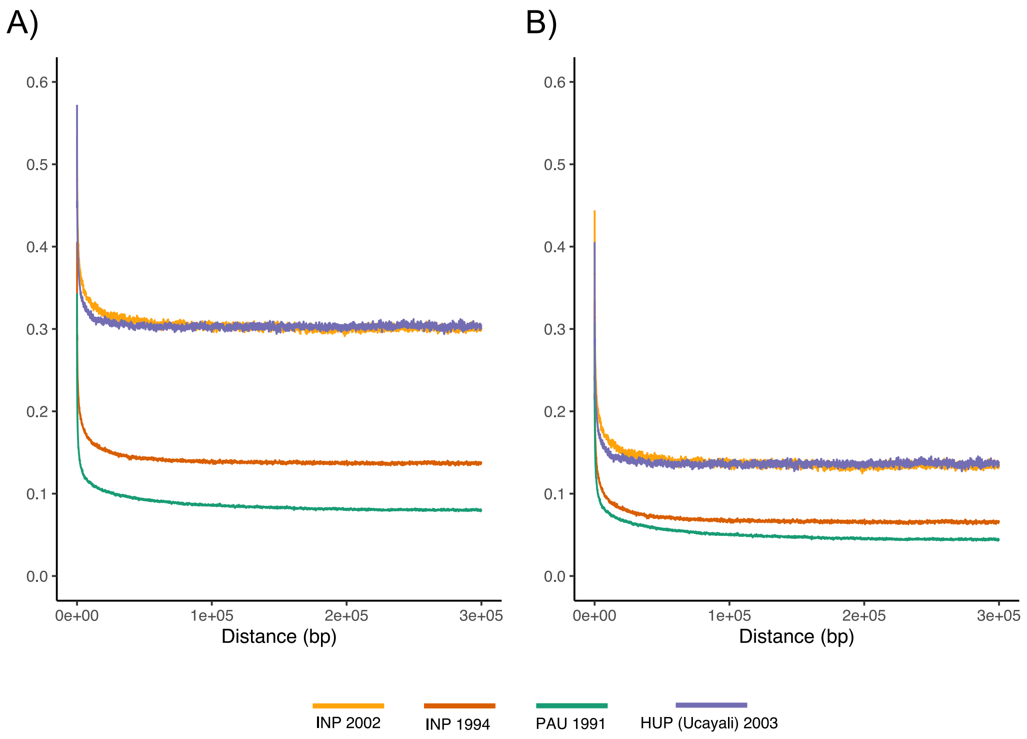

Supplementary Figure 3. Linkage Disequilibrium decay plots after correction for population structure and spatio-temporal Wahlund effects. **A)** Uncorrected for sample size. **B)** Corrected for sample size (r2 - 1/(2n)) [20]**.**

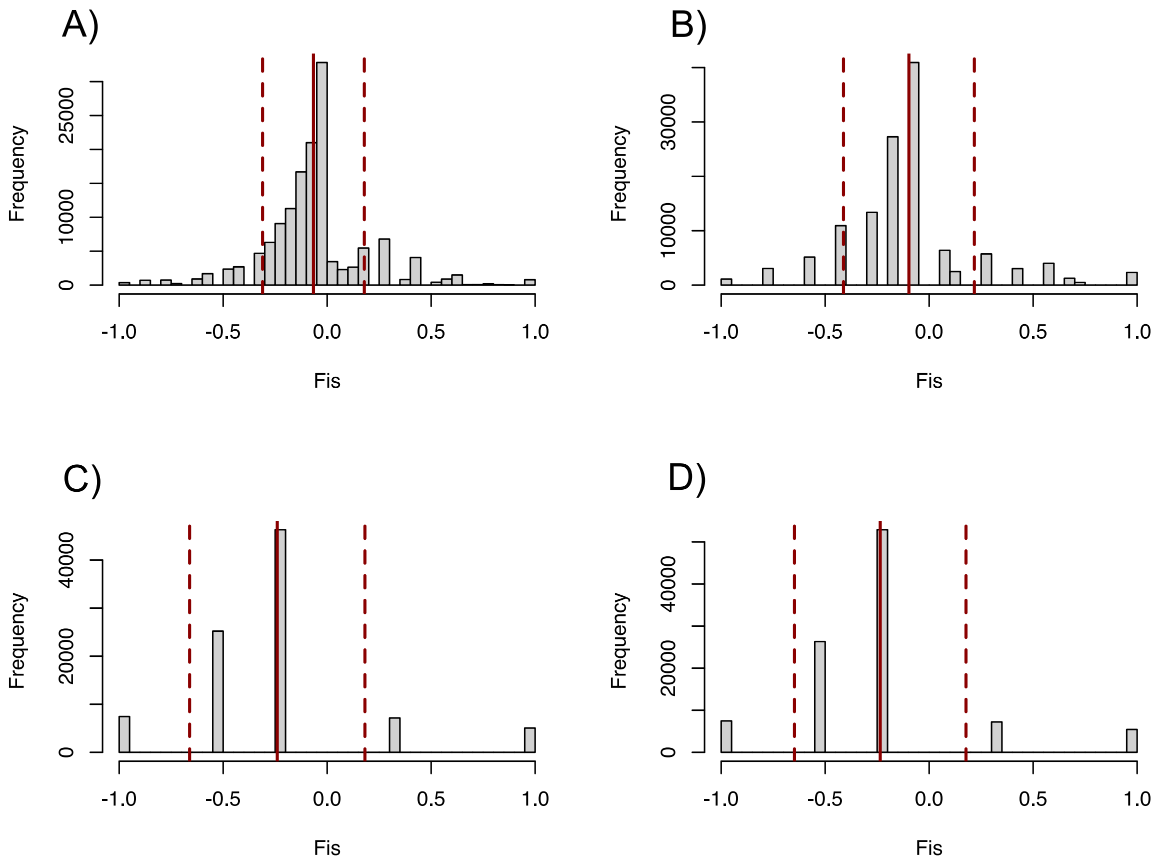

Supplementary Figure 4. Fis distributions after correction for population structure and spatio-temporal Wahlund effects. **A)** Individuals from PAU sampled in 1991 (N=14). **B)** Individuals from INP sampled in 1994 (N=7). **C)** Individuals from INP sampled in 2002 (N=3). **D)** Individuals from HUP (Ucayali) sampled in 2003 (N=3). Solid red lines depict the mean Fis value for each population. Dashed red lines represent the mean's standard deviation.

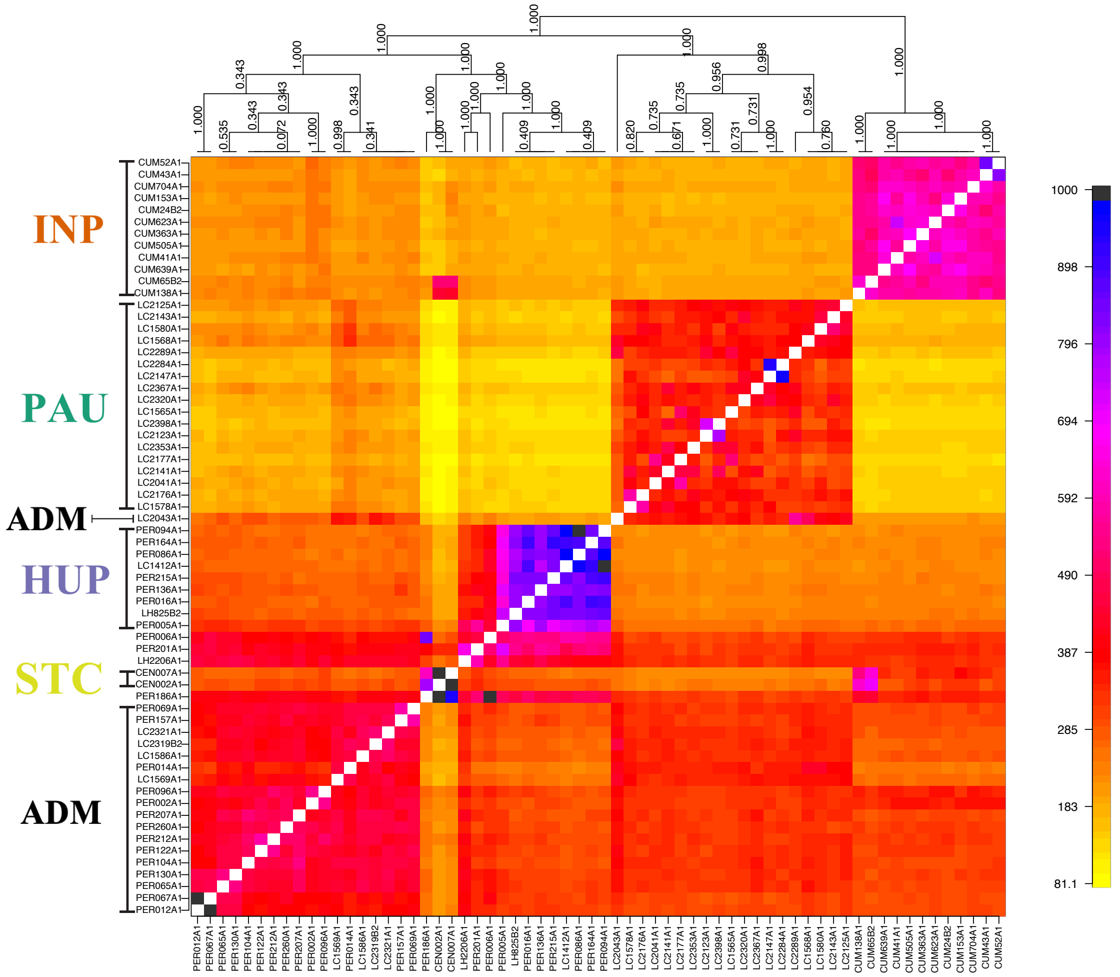

Supplementary Figure 5. Co-ancestry matrix, as inferred by fineSTRUCTURE, depicting the pairwise number of received (rows) and donated (columns) haplotype segments between two parasite genomes. Color key on the right shows the amount of shared haplotype segments and is capped on 1000 for visibility reasons. Individuals are ordered according to the fineSTRCUTURE clustering outcome (above matrix) and dashed accolades indicate the three ancestral parasite groups (INP, HUP, PAU) and the two main groups of admixed parasites (ADM, STC); the remainder of the parasites were of uncertain ancestry (UNC).

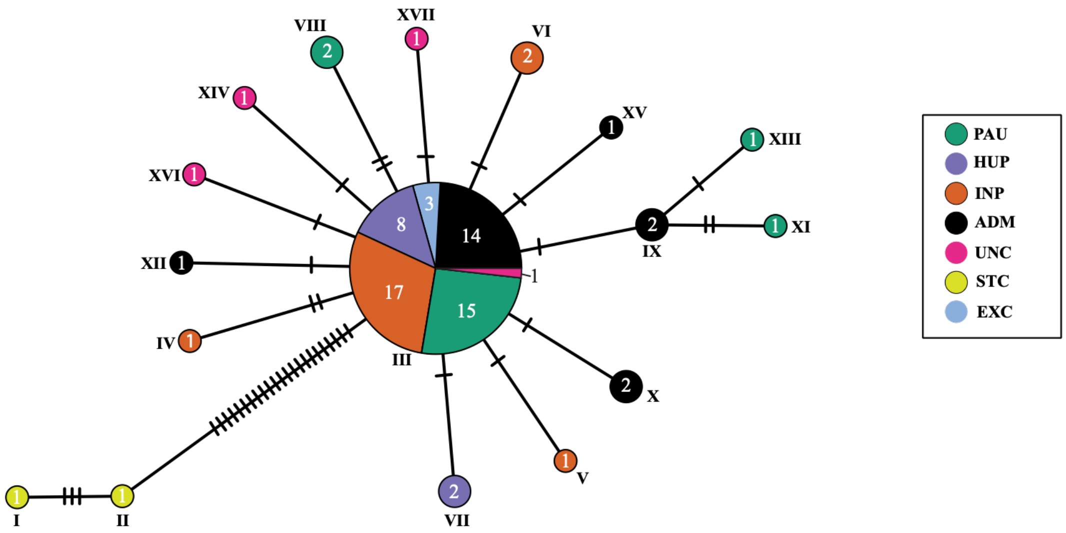

Supplementary Figure 6. TCS Haplotype network with PopART based on 53 high-quality SNPs identified within the coding region of the haploid mitochondrial maxicircle. A total of 17 haplotypes (shown with Roman numbers) were identified within our set of 79 *L. braziliensis* isolates from Peru and Bolivia. The size of the circles represent the number of sequences that represent a given haplotype; this number is also written within each circle. Colors indicate the six groups of parasites as identified with ADMIXTURE, fineSTRUCTURE and PCAdmix using genome-wide SNPs; the EXC group indicates the three isolates that were excluded from population structure analyses. The dominant haplotype III is represented by 58 maxicircle sequences (73.4% of the 79 included sequences) and is found in four of the five groups. Black bars represent the number of mutations between two haplotypes.

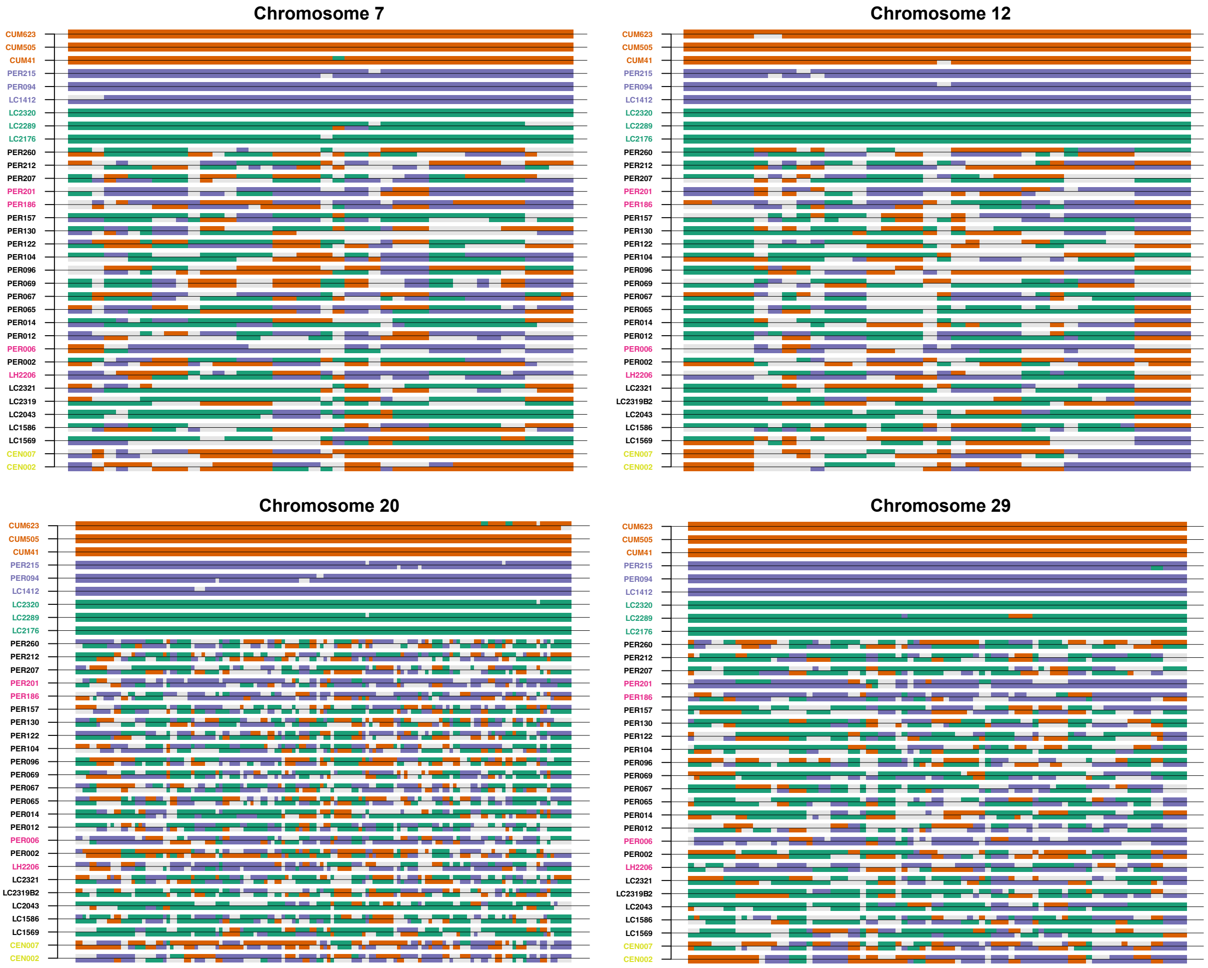

Supplementary Figure 7. PCAdmix local ancestry assignment to PAU, INP and HUP source populations of 19 ADM isolates, 4 UNC isolates, 2 STC isolates and three randomly selected isolates from each source population. Ancestry was assigned in windows of 30 SNPs along each chromosome (here only chromosomes 7, 12, 20 and 29 are shown).

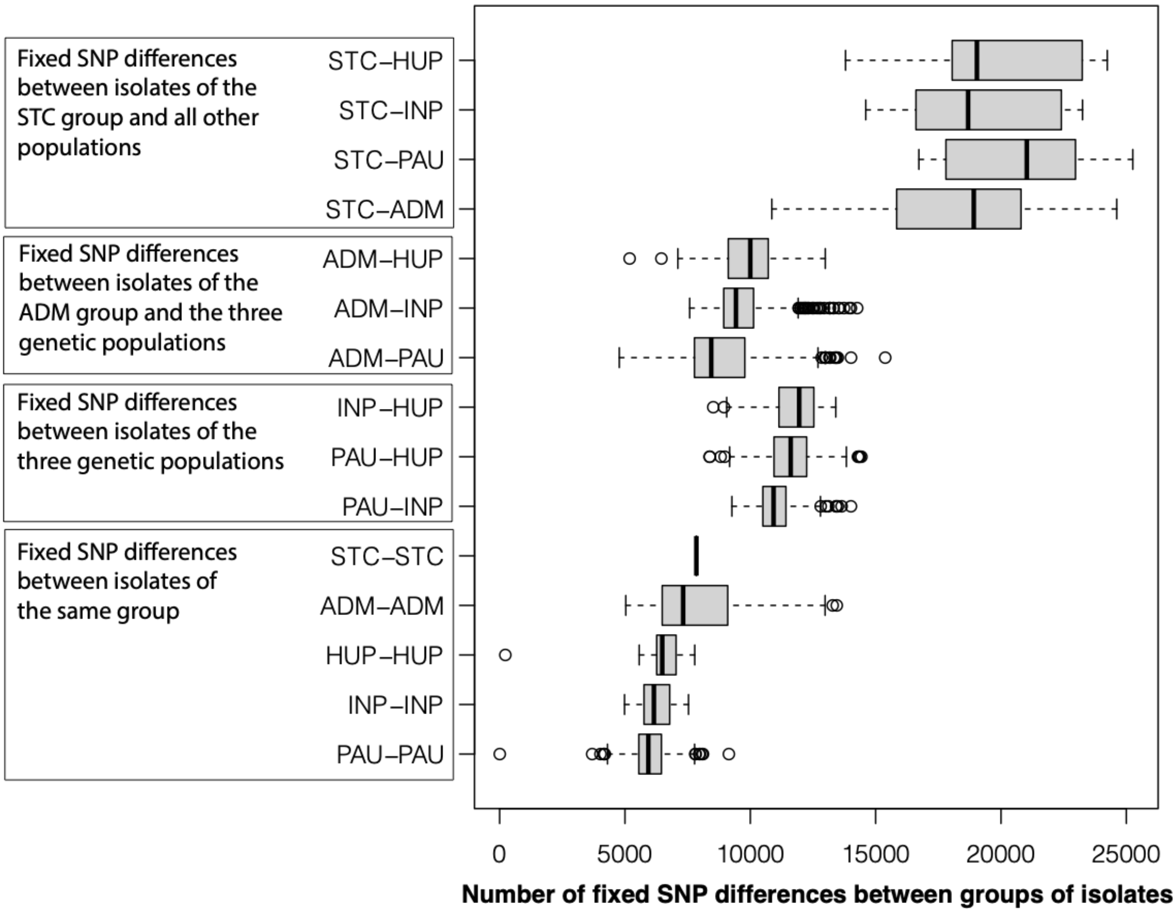

Supplementary Figure 8. Number of fixed SNP differences between isolates of the same group or between isolates of different groups

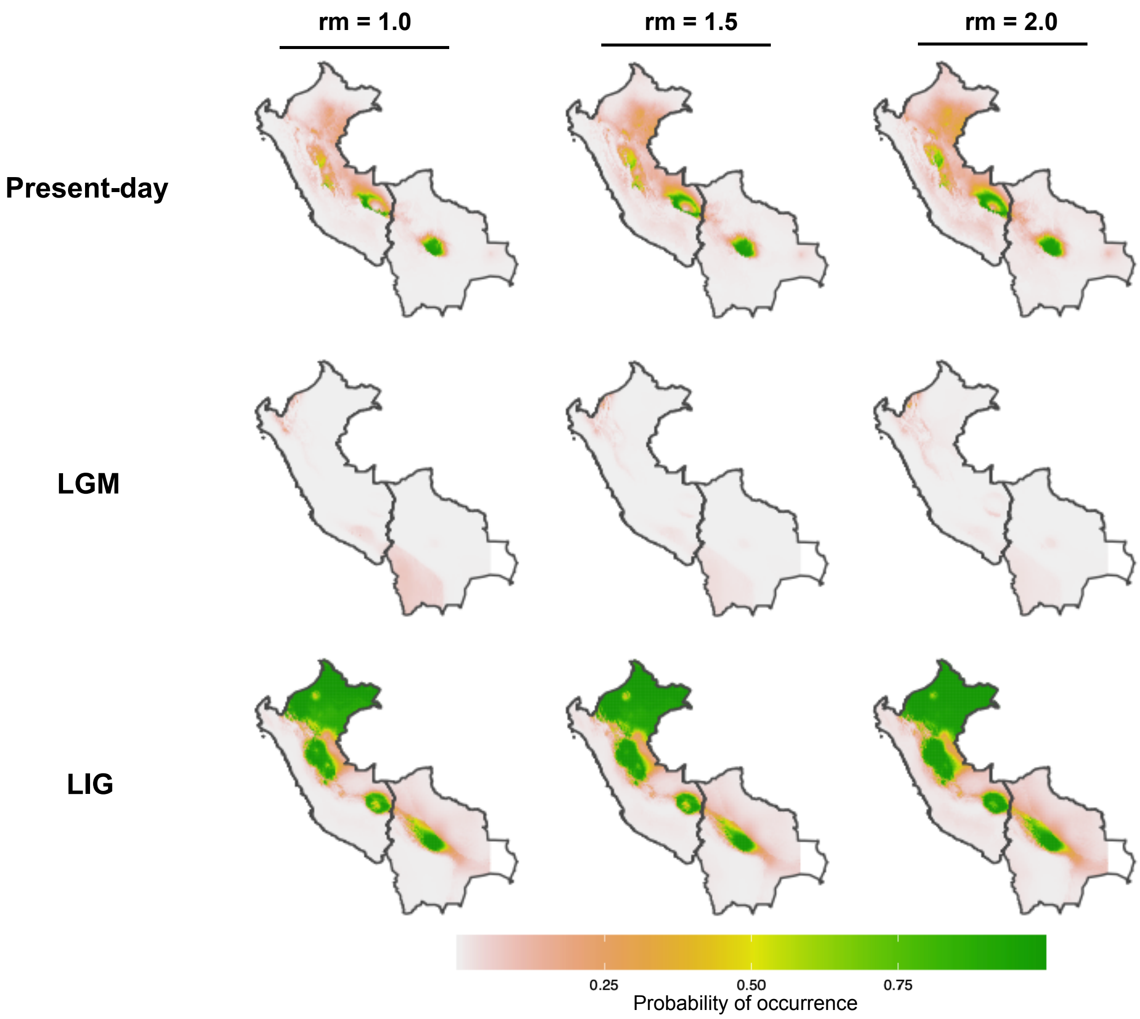

Supplementary Figure 9. Ecological niche models projected on Present-day, LGM and LIG bioclimatic data (bio2, bio3, bio14, bio18) with different regularization values (rm = 1, 1.5 and 2). The continuous-scale legend represents habitat suitability (probability of occurrence). Country-level data for Peru and Bolivia, including administrative boundaries, were available from: http://www.diva-gis.org/Data

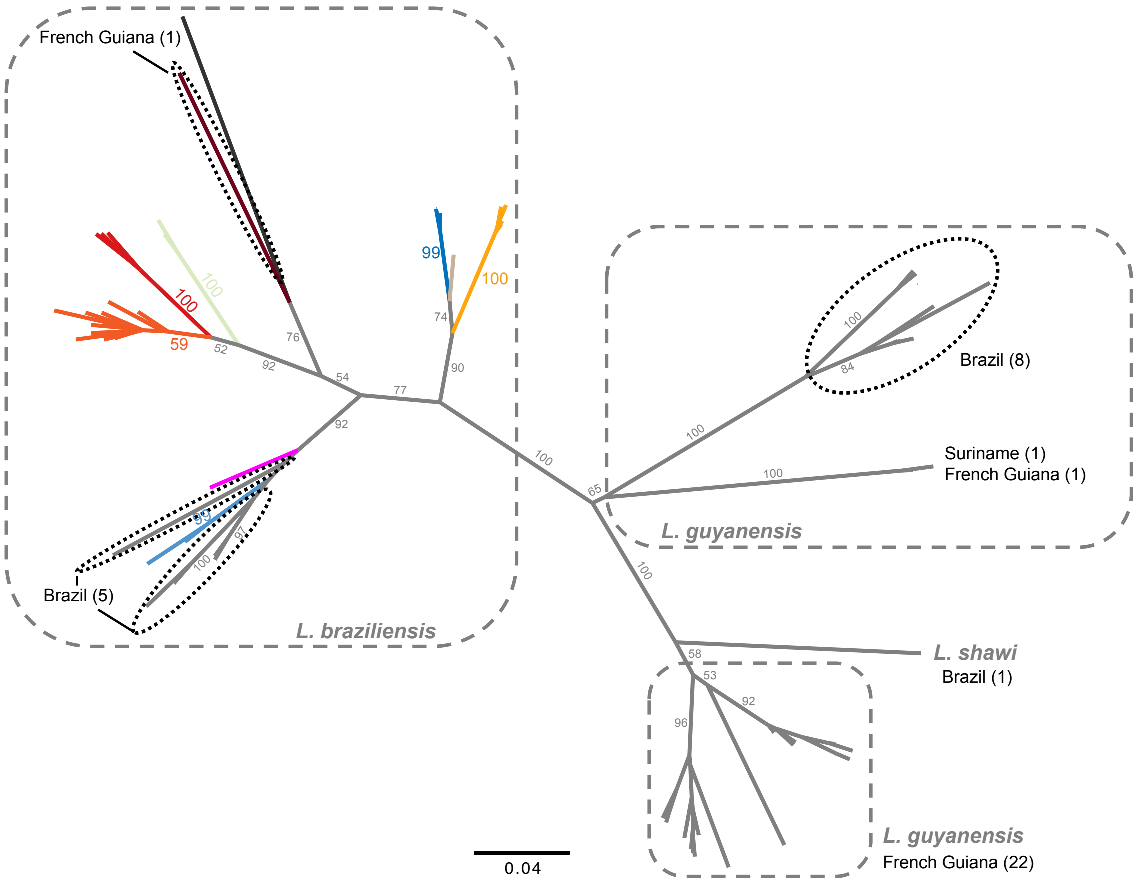

Supplementary Figure 10. Maximum likelihood tree based on partial LRV1 sequences (756bp) from *L. braziliensis*, *L. guyanensis* and *L. shawi* originating from Peru, Bolivia, Brazil, French Guiana and Suriname [21–24]. Colored lineages in *L. braziliensis* correspond to the LRV1 lineages described in this study (Fig. 6A). Bootstrap percentages are given for each node.

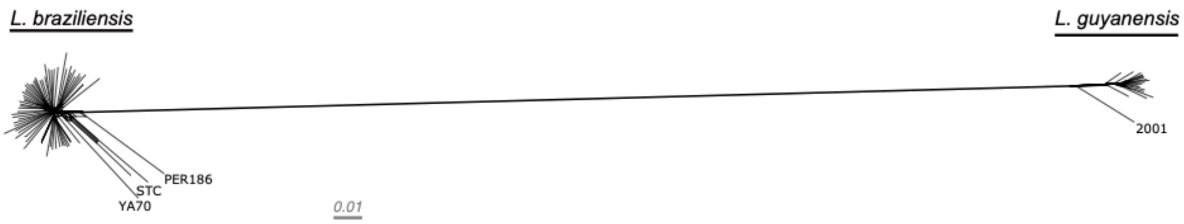

Supplementary Figure 11. A phylogenetic network, inferred with SPLITSTREE, based on uncorrected p-distances between 77 *L. brazilienis* and 19 *L. guyanensis* isolates typed at 7,571 bi-allelic SNPs. *Leishmania* genotypes were based on SNPs called with GATK Haplotypecaller across our set of 79 *L. braziliensis* genomes and a set of 20 publicly available *L. guyanensis* genomes (see methods). Despite the relatively low coverage of the *L. guyanensis* genomes (median 3x, min 1x, max 4x), genotyping recovered a total of 7,571 high-quality bi-allelic SNPs called across 99 *Leishmania* genomes.

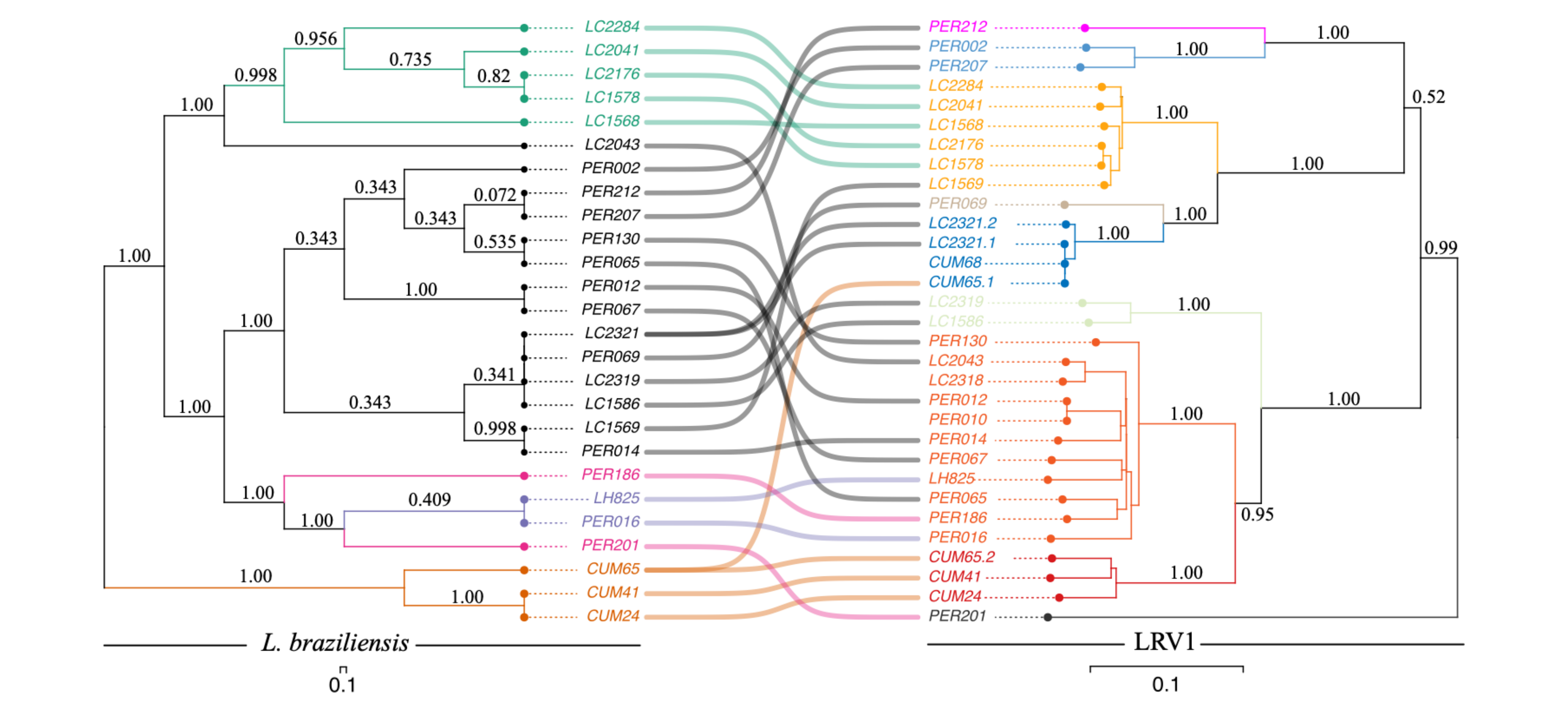

Supplementary Figure 12. A phylogenetic tangle plot showing patterns of virus-parasite co-divergence and intraspecific host-switching between *L. braziliensis* and its respective LRV1 lineages. (left) fineSTRUCTURE tree of LRV1+ *L. braziliensis* isolates (adapted from Figure 1b). Tree branches, tips and tree links are coloured according to the inferred parasite groups. (right) Maximum likelihood tree of LRV1 (adapted from Figure 4b). Tree branches and tips are coloured according to the nine lineages described in this study.

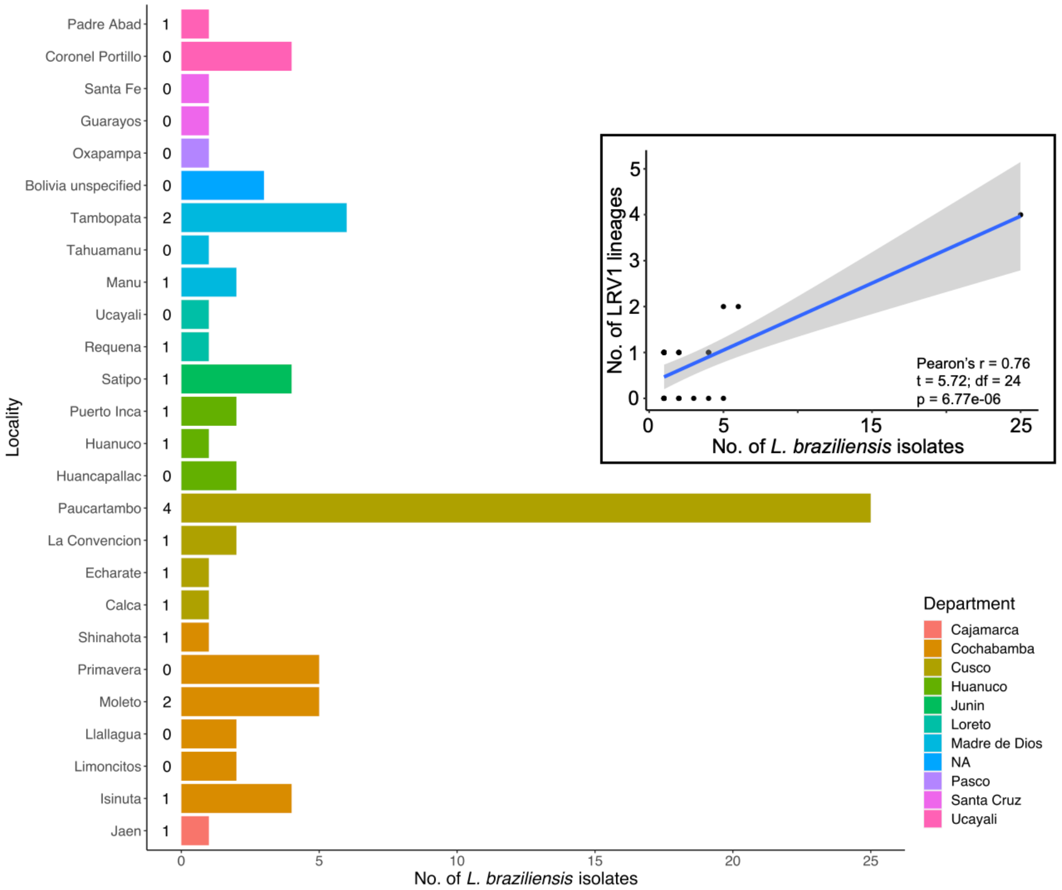

Supplementary Figure 13. Barplot shows the number of isolates per sampling locality. Number on the left of each bar shows the number of LRV1 lineages that were identified per locality. Bars are coloured according to the Department. Inset reveals the number of LRV1 lineages versus the number of LRV1 isolates that were recovered in a given locality.

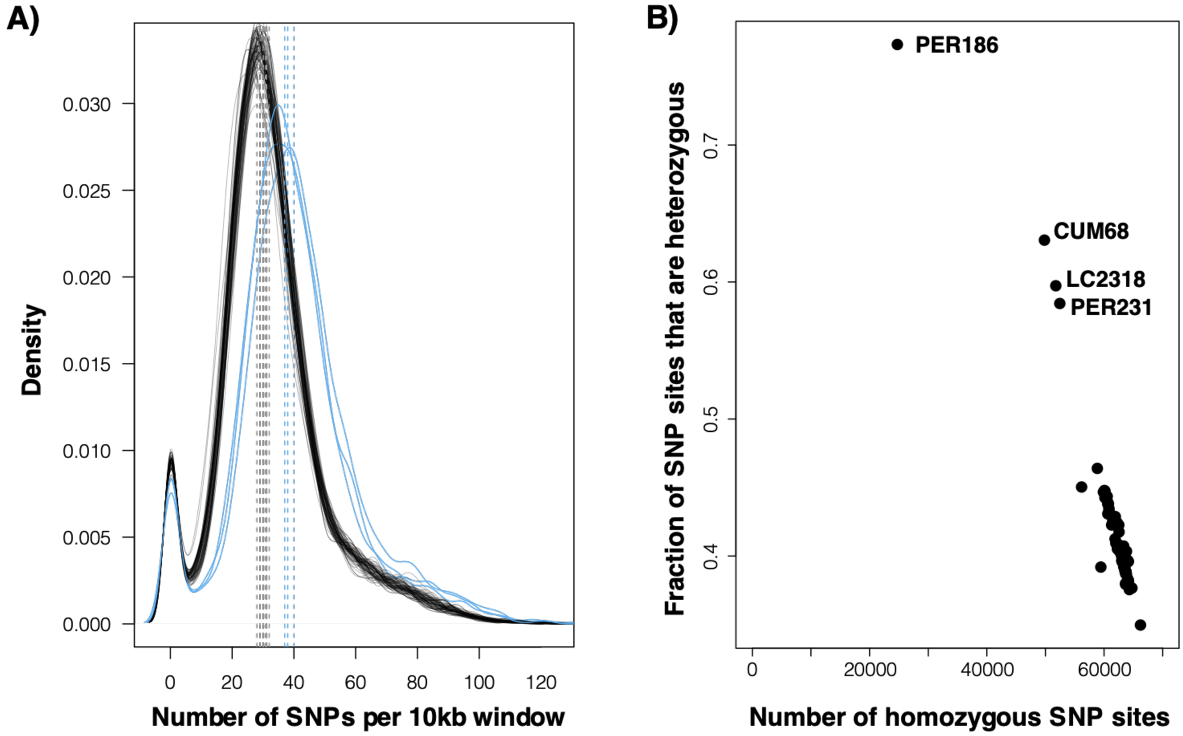

Supplementary Figure 14. A) Kernel density plots of the number of SNPs per 10kb window for each of the 79 parasite genomes. The median number of SNPs per 10kb window is indicated with gray vertical dashed lines and ranges between 28 and 32 SNPs for the majority of isolates. Three isolates showed slightly larger SNP densities (indicated with blue lines in the plot): 37 SNPs in PER231, 38 SNPs in LC2318 and 40 SNPs in CUM68. **B)** Fraction of SNP sites that are heterozygous versus the number of homozygous SNP sites in each of the 79 genomes.

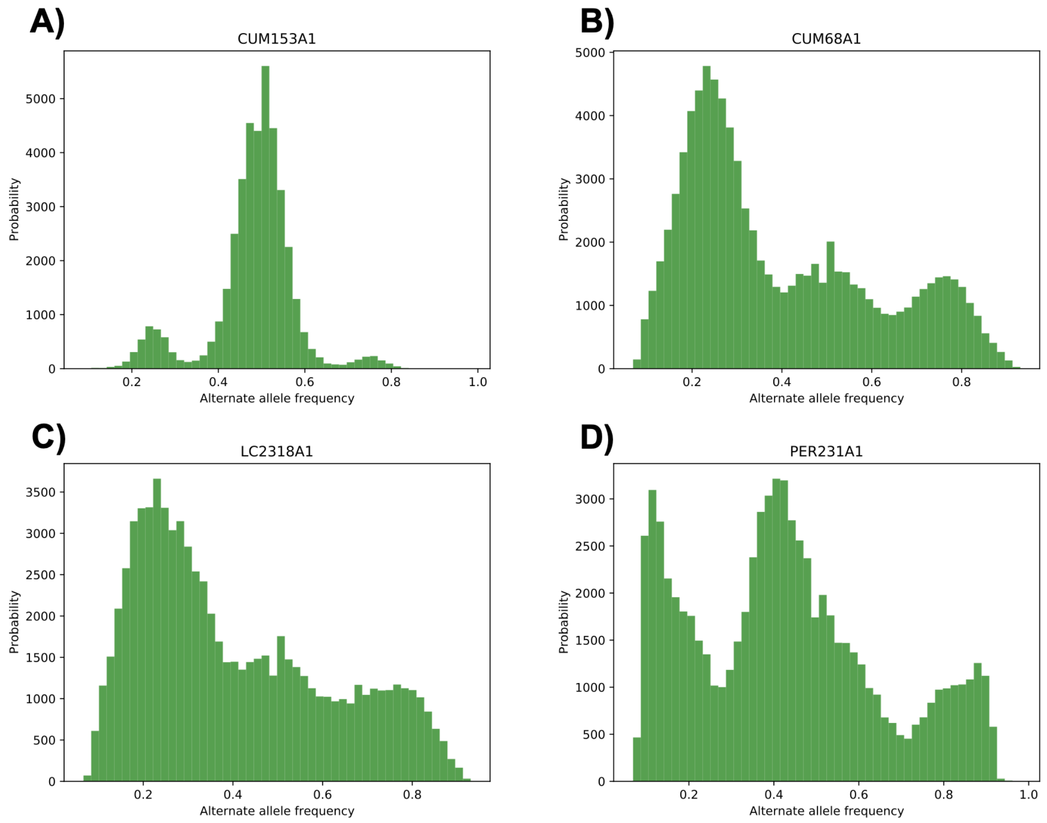

Supplementary Figure 15. Genome-wide distribution of alternate allele read depth frequencies at heterozygous sites. **(A)** Example of a largely diploid individual (CUM153) with allele frequencies centered around 0.5, which was observed for 77/79 genomes included in this study. **(B-C)** Two isolates (CUM68 and LC2318) were symptomatic of tetraploidy, with modes of allele frequencies equal to 0.25, 0.5 and 0.75. **(C)** One isolate (PER231) showed a skewed distribution, suggesting that it may be the result of a mixed infection or contamination.

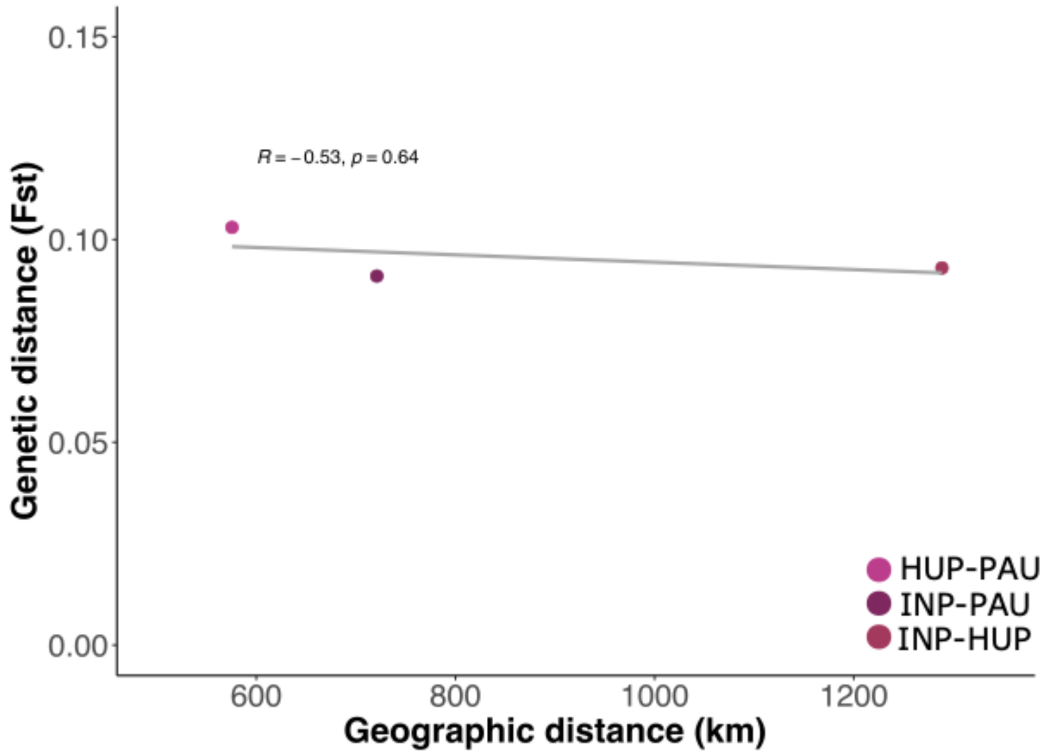

Supplementary Figure 16. Linear regression of the interdeme great-circle distance (km) and the Weir & Cockerham's Fst of the three ancestral *L. braziliensis* components.

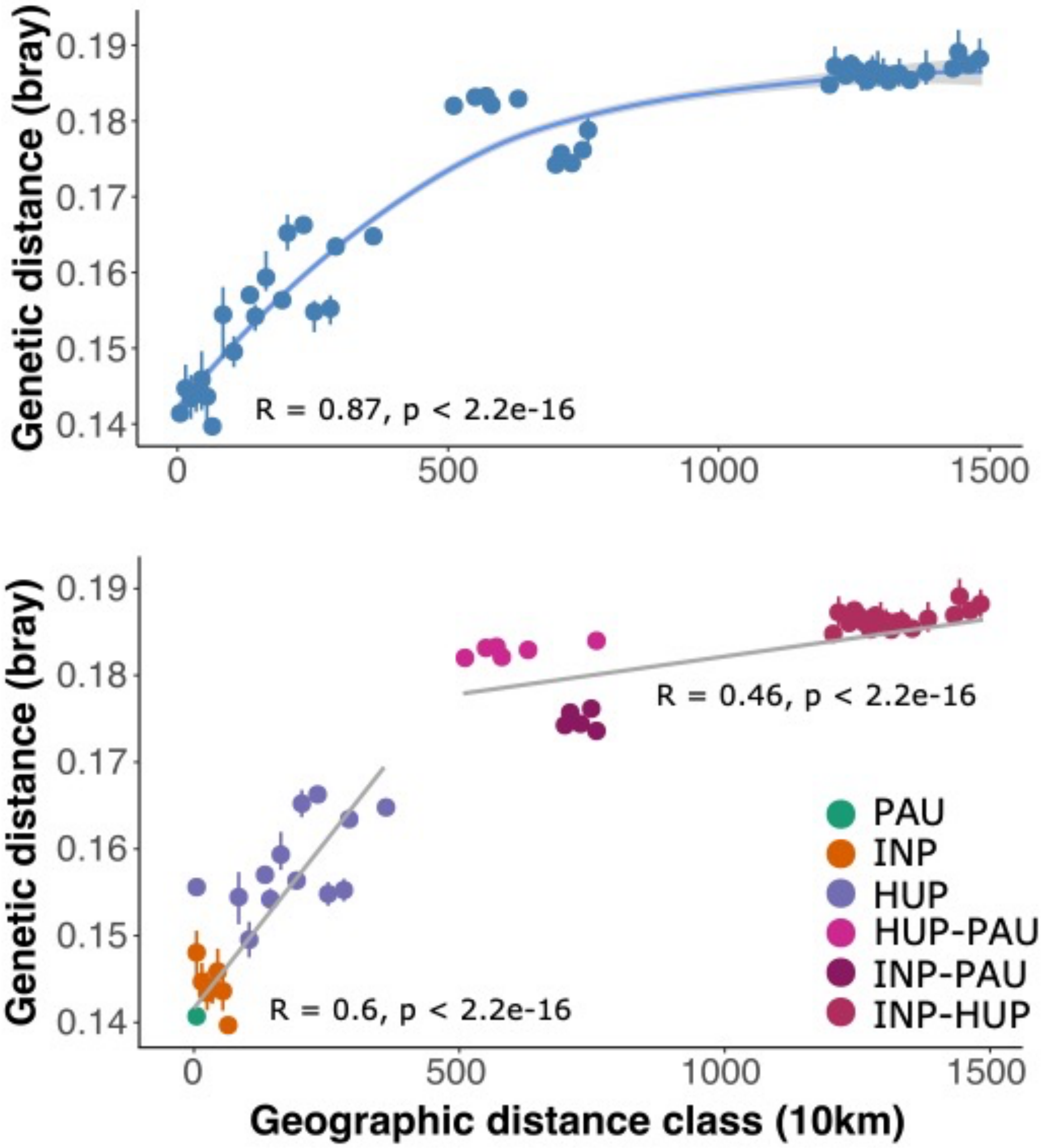

Supplementary Figure 17. Regressions of the inter-individual great-circle distance (km) and pairwise genetic distance (Bray-Curtis dissimilarity of SNP genotypes) revealing a case-IV IBD pattern. a) Loess regression for all individuals with a loess value of 1.2. b) Linear regression of intra- and inter-population genetic distance vs. geographic distance, separately.

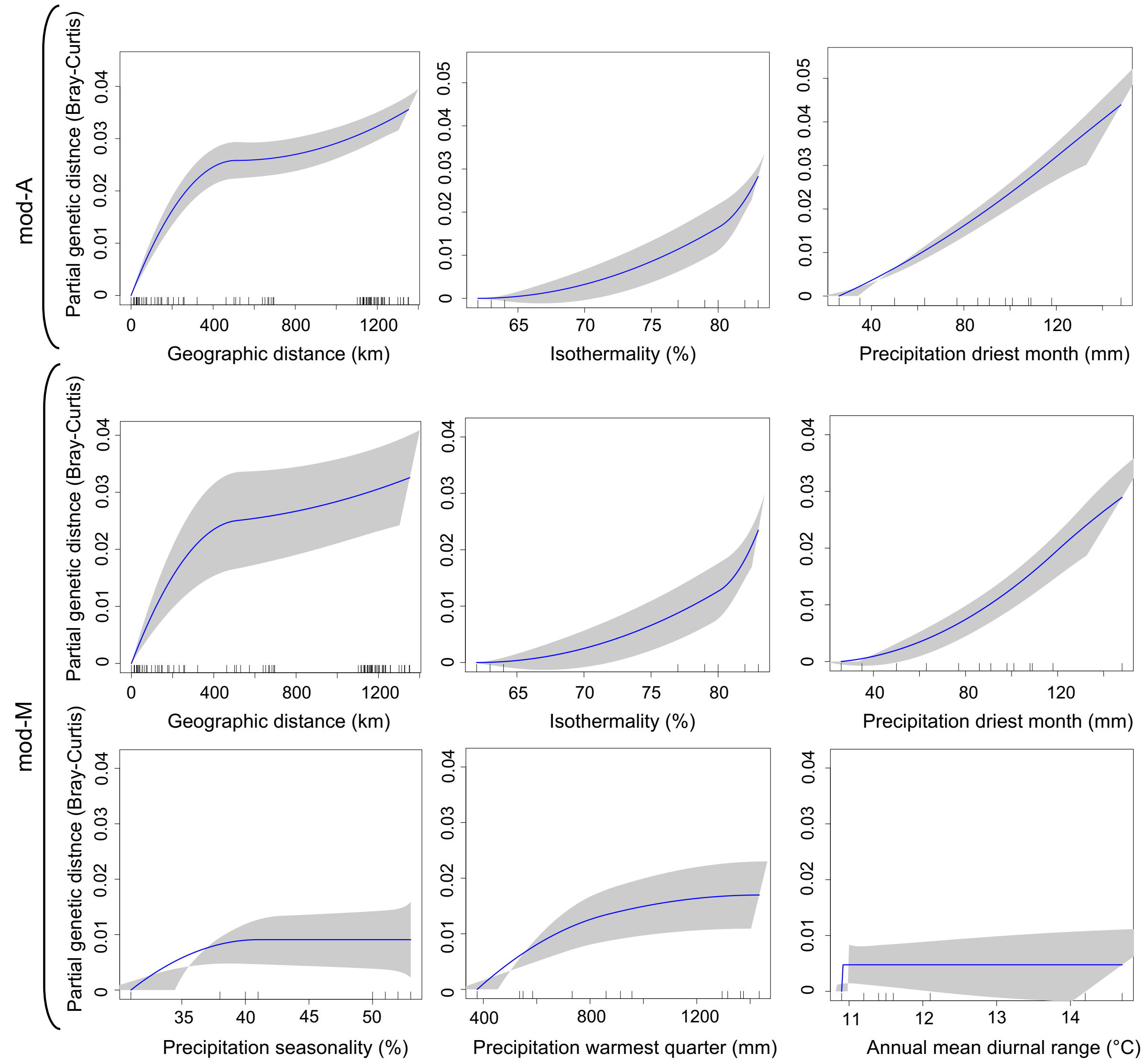

Supplementary Figure 18. I-spline response curves for each variable included in the different generalized dissimilarity models (mod-A & mod-M). The maximum curve height represents the amount of genetic variability the variable explains (i.e. the variable importance). The curves' slope indicates the degree of the explained genomic dissimilarity along the spatial or environmental gradient, meaning a steeper slope represents greater dissimilarity between two points while a shallower slope suggests less variability among the two points.

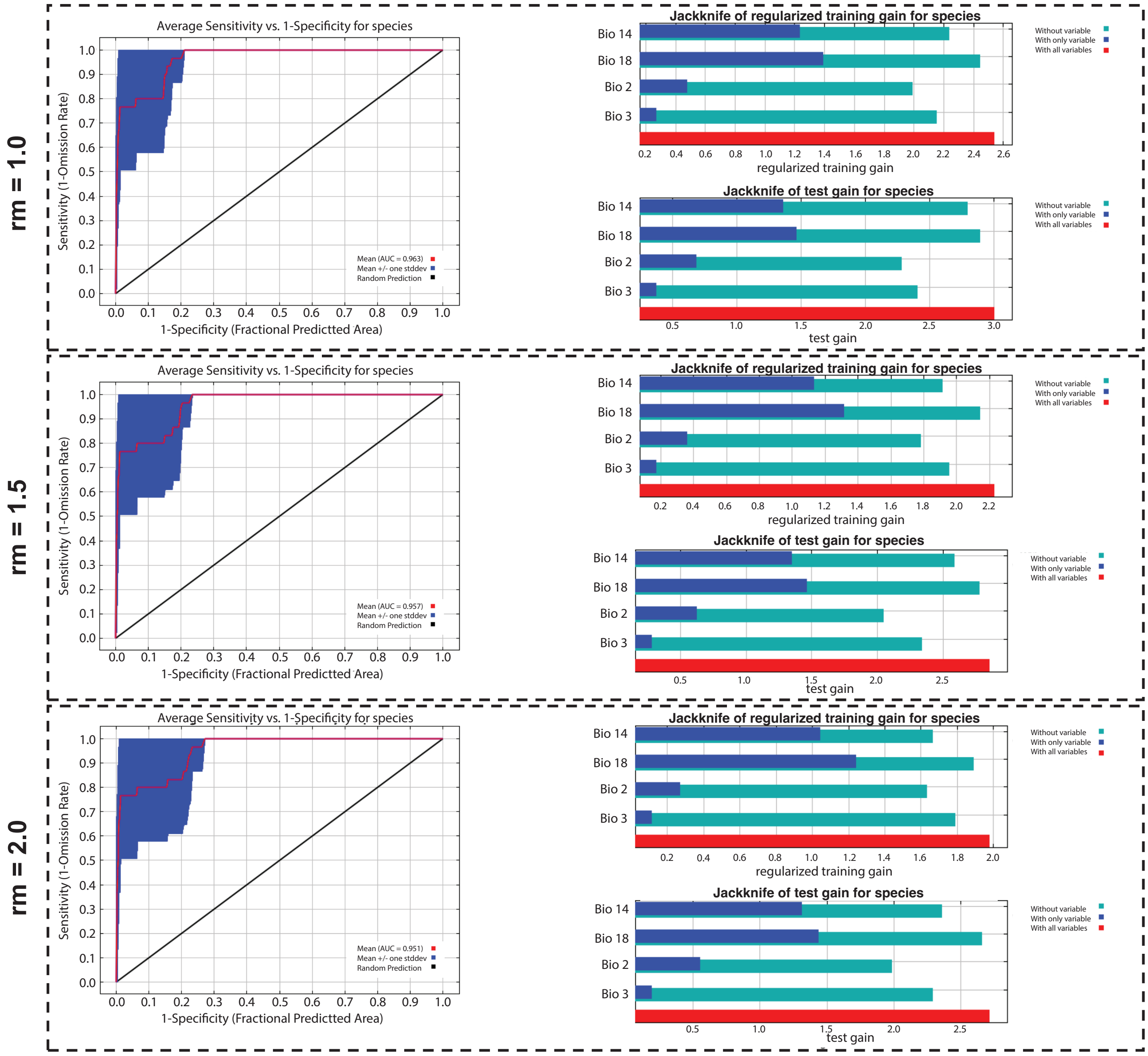

Supplementary Figure 19: Model performance and Variable contribution for the three ecological niche models (rm = 1 - 1.5 - 2). (Left) ROC-analysis of the respective model based on 10 cross-validation replicates. (Right) Jackknife tests for training (upper) and testing (lower) gain of each bioclimatic variable included in the model. Bio3 = isothermality; bio14 = precipitation of the driest month; bio18 = precipitation of the warmest quarter; bio15 = precipitation seasonality; and bio2 = annual mean diurnal range.

Supplementary references

1. Fick, S. E. & Hijmans, R. J. WorldClim 2: new 1-km spatial resolution climate surfaces for global land areas. *Int. J. Climatol.* **37**, 4302–4315 (2017).
2. Padgham, M. & Sumner, M. D. *geodist: Fast, Dependency-Free Geodesic Distance Calculations. R package version 0.0.7*. (2021).
3. Danecek, P. *et al.* The variant call format and VCFtools. *Bioinformatics* **27**, 2156–2158 (2011).
4. Oksanen, J. *et al. Vegan Community Ecology Package Version 2.5-7*. (2020).
5. Capblancq, T. & Forester, B. R. Redundancy analysis: A Swiss Army Knife for landscape genomics. *Methods Ecol. Evol.* **12**, 2298–2309 (2021).
6. Fitzpatrick, M., Mokany, K., Manion, G., Nieto-Lugilde, D. & Ferrier, S. *gdm: Generalized Dissimilarity Modeling*. (2022).
7. Ferrier, S., Manion, G., Elith, J. & Richardson, K. Using generalized dissimilarity modelling to analyse and predict patterns of beta diversity in regional biodiversity assessment. *Divers. Distrib.* **13**, 252–264 (2007).
8. Mokany, K., Ware, C., Woolley, S. N. C., Ferrier, S. & Fitzpatrick, M. C. A working guide to harnessing generalized dissimilarity modelling for biodiversity analysis and conservation assessment. *Glob. Ecol. Biogeogr.* **31**, 802–821 (2022).
9. Karger, D. N., Nobis, M. P., Normand, S., Graham, C. H. & Zimmermann, N. E. CHELSA-TraCE21k – high-resolution (1 km) downscaled transient temperature and precipitation data since the Last Glacial Maximum. *Clim. Past Discuss.* 1–27 (2023).
10. Brown, J. L., Hill, D. J., Dolan, A. M., Carnaval, A. C. & Haywood, A. M. PaleoClim, high spatial resolution paleoclimate surfaces for global land areas. *Scientific Data* **5**, (2018).
11. Otto-Bliesner, B. L., Marshall, S. J., Overpeck, J. T., Miller, G. H. & Hu, A. Simulating Arctic climate warmth and icefield retreat in the last interglaciation. *Science* **311**, 1751–1753 (2006).
12. Hijmans, R. J., Phillips, S., Leathwick, J., Elith, J. & Hijmans, M. R. J. Package 'dismo'. *Circles* **9**, 1–68 (2017).
13. Phillips, S. J., Anderson, R. P. & Schapire, R. E. Maximum entropy modeling of species geographic distributions. *Ecol. Modell.* **190**, 231–259 (2006).
14. Van den Broeck, F. *et al.* Ecological divergence and hybridization of Neotropical Leishmania parasites. *Proc. Natl. Acad. Sci. U. S. A.* **117**, 25159–25168 (2020).
15. Rogers, M. B. *et al.* Chromosome and gene copy number variation allow major structural change between species and strains of Leishmania. *Genome Res.* **21**, 2129–2142 (2011).
16. Walker, B. J. *et al.* Pilon: an integrated tool for comprehensive microbial variant detection and genome assembly improvement. *PLoS One* **9**, e112963 (2014).
17. Fritz, A. *et al.* Haploflow: strain-resolved de novo assembly of viral genomes. *Genome Biol.* **22**, 212 (2021).
18. Imamura, H. *et al.* Evolutionary genomics of epidemic visceral leishmaniasis in the Indian subcontinent. *Elife* **5**, (2016).
19. Franssen, S. U. *et al.* Global genome diversity of the Leishmania donovani complex. *Elife* **9**, (2020).
20. Shin, D., Kim, S.-H., Park, J., Lee, H.-K. & Song, K.-D. Extent of linkage disequilibrium and effective population size of the Landrace population in Korea. *Asian-australas. J. Anim. Sci.* **31**, 1078–1087 (2018).

21. Tirera, S. *et al.* Unraveling the genetic diversity and phylogeny of Leishmania RNA virus 1 strains of infected Leishmania isolates circulating in French Guiana. *PLoS Negl. Trop. Dis.* **11**, e0005764 (2017).
22. Widmer, G., Comeau, A. M., Furlong, D. B., Wirth, D. F. & Patterson, J. L. Characterization of a RNA virus from the parasite Leishmania. *Proc. Natl. Acad. Sci. U. S. A.* **86**, 5979–5982 (1989).
23. Scheffter, S., Widmer, G. & Patterson, J. L. Complete sequence of Leishmania RNA virus 1-4 and identification of conserved sequences. *Virology* **199**, 479–483 (1994).
24. Cantanhêde, L. M. *et al.* New insights into the genetic diversity of Leishmania RNA Virus 1 and its species-specific relationship with Leishmania parasites. *PLoS One* **13**, e0198727 (2018).
