# Supplementary material for: Diversity and dissemination of viruses in pathogenic protozoa
Source: Nat Commun. 2023 Dec 15;14:8343. doi: 10.1038/s41467-023-44085-2 (PMC10724245; doi:10.1038/s41467-023-44085-2)
Supplement: Supplementary file 3 — Description of Additional Supplementary Files [file 41467_2023_44085_MOESM3_ESM.pdf]

Description of Additonal Supplementary Files

Supplementary Data 1 List of 79 Lb isolates included in the study.

Supplementary Data 2 Gene copy numbers as estimated using normalized read depths per coding DNA sequence, grouped per orthologous group.

Supplementary Data 3 Information on the inbreeding coefficient (Fis), expected heterozygosity (He) and observed heterozygosity (Ho) for four subsets of isolates close in time and space.

Supplementary Data 4 Information and details about the clonal groups. Isolates within each group can be found in Supp. Data 1.

Supplementary Data 5 Spatio-environmental variables included in RDA and GDM analyses. The first two columns represent the decimal coordinates of each Lb isolate. Bio1-19 represent the 19 bioclimatic variables from the WorldClim2 database. Bio1= Annual mean temperature; Bio2= Mean diurnal range; Bio3= Isothermality (Bio2/Bio7)*100); Bio4= Temperature seasonality; Bio5= Max. temperature of warmest month; Bio6= Min. temperature of coldest month; Bio7= Temperature annual range; Bio8=Mean temperature of wettest quarter; Bio9= Mean temperature of the driest quarter; Bio10= Mean temperature of of the warmest quarter; Bio11= Mean temperature of the coldest quarter; Bio12=Annual precipitation; Bio13=Precipitation of wettest month; Bio14= Precipitation of driest month; Bio15= Precipitation seasonality; Bio16= Precipitation of wettest quarter; Bio17=Precipitation of driest quarter; Bio18= Precipitation of warmest quarter; Bio19= Precipitation of coldest quarter. Bio1-11: unit = °C*10; Bio12-19: unit = mm; Bio3 & Bio 15: unit = %.

Supplementary Data 6 Results of the RDA-based variable selection to reduce overfitting and multicollinearity. The upper sub-table represents the bioclimatic variables selected by the automated selection approach. The lower sub-table represents the bioclimatic variables selected by the manual selection approach. Tests (two sided) for variable selection were based on ANOVA like permutation tests for RDA analyses as implemented in the vegan R package (see methods).

Supplementary Data 7 RDA results for both variable selection approaches. The upper sub-table represents the RDA results for the automated variable selection approach (mod-a). The lower sub-table represents the RDA results obtained by the manual variable selection approach (mod-m). Tests (two sided) were RDA analyses accompanied by ANOVA like permutation tests for RDA analyses as implemented in the vegan R package (see methods).

Supplementary Data 8 Kruskal-Wallis test and pairwise Dunn's test comparing the differences among the geographic ranges of the inferred parasite groups. The upper gray box presents the results from the Kruskal-Wallis test. The accompanying table represents the Z-statistic (above diagonal) and Benjamini-Hochberg (BH) corrected p-values (below diagonal) of the pairwise Dunn's tests. Sample sizes are given in the diagonal.

Supplementary Data 9 Information on the LRV1 assembled genomes.

Supplementary Data 10 Pairwise 'raw' genetic distance among LRV1 genomes, representing the proportion of sites that are dissimilar between two genomes.

Supplementary Data 11 Diversity metrics within and between the nine LRV1 lineages. The diagonal (dark grey): the nucleotide diversity within each viral lineage. Above the diagonal (light grey): Pairwise nucleotide diversity between viral lineages. Below diagonal (white): Pairwise Fst between viral lineages.

Supplementary Data 12 Kruskal-Wallis test and pairwise Dunn's test comparing the differences between the diffusion coefficients of the whole LRV1 phylogeny and lineage-excluded partitions. The upper gray box presents the results from the Kruskal-Wallis test. The accompanying table represents the Z-statistic (above diagonal) and Benjamini-Hochberg (BH) corrected p-values (below diagonal) of the pairwise Dunn's tests. Sample sizes (i.e. diffusion coefficient estimates) are given in the diagonal.

Supplementary Data 13 Fisher's exact tests (two sided) comparing viral prevalences among parasite groups. Upper gray box presents the overall Fisher's exact test. The values below the diagonal represent the Benjamini-Hochberg (BH) corrected p-values of the pairwise Fisher's exact tests. The diagonal hold two lineage diversity indices: the Shannon diversity and the lineage richness (in brackets). Sample sizes are given in the table columns in brackets.

Supplementary Data 14 Cross validation error values for the different ADMIXTURE models (K = 1-10).

Supplementary Data 15 List of 80 variants (SNPs or INDELs) with a deleterious impact on genes in Lb genomes, as revealed by the program SNPEFF. For each variant, allele frequencies are summarized per Lb population (columns K-P) as inferred with population genomic analyses. Allele frequencies were also estimated for the group of LRV+ isolates (column Q) and LRV- isolates (column R). Genotypes are given for each of the 76 Lb isolates (columns S-CP), and were coded as 0 (homozygous for the reference allele), 1 (heterozygous) or 2 (homozygous for the alternate allele).

Supplementary Data 16 Weir & Cockerham's Fst among the three distinct Lb populations estimated in 50kb windows.

Supplementary Data 17 inter-individual Bray-Curtis dissimilarity among isolates of the three distinct Lb populations.

Supplementary Data 18 Variable Selection for the ecological niche models based on the preceding RDA and GDM analyses. The balues below the diagonal depict the correlation coefficient between the pairs of bioclimatic variables. Correlation coefficients that exceeded |0.7| are depicted in bold. The diagonal (gray) shows the variant inflation factors including (outside brackets) and exclusing bio15 (inside brackets).

Supplementary Data 19 Model performance statistics and variable contributions for the three environmental niche models.
