# Supplementary material for: Diversity and dissemination of viruses in pathogenic protozoa
Source: Nat Commun. 2023 Dec 15;14:8343. doi: 10.1038/s41467-023-44085-2 (PMC10724245; doi:10.1038/s41467-023-44085-2)
Supplement: Supplementary file 5 — Reporting Summary [file 41467_2023_44085_MOESM5_ESM.pdf]

nature portfolio

Corresponding author(s): Dr. Frederik Van den Broeck
Last updated by author(s): Nov 16, 2023

Reporting Summary

Nature Portfolio wishes to improve the reproducibility of the work that we publish. This form provides structure for consistency and transparency in reporting. For further information on Nature Portfolio policies, see our Editorial Policies and the Editorial Policy Checklist.

Statistics

For all statistical analyses, confirm that the following items are present in the figure legend, table legend, main text, or Methods section.

n/a	Confirmed	
☐	☒	The exact sample size (*n*) for each experimental group/condition, given as a discrete number and unit of measurement
☒	☐	A statement on whether measurements were taken from distinct samples or whether the same sample was measured repeatedly
☐	☒	The statistical test(s) used AND whether they are one- or two-sided<br>*Only common tests should be described solely by name; describe more complex techniques in the Methods section.*
☐	☒	A description of all covariates tested
☐	☒	A description of any assumptions or corrections, such as tests of normality and adjustment for multiple comparisons
☐	☒	A full description of the statistical parameters including central tendency (e.g. means) or other basic estimates (e.g. regression coefficient) AND variation (e.g. standard deviation) or associated estimates of uncertainty (e.g. confidence intervals)
☐	☒	For null hypothesis testing, the test statistic (e.g. *F*, *t*, *r*) with confidence intervals, effect sizes, degrees of freedom and *P* value noted<br>*Give P values as exact values whenever suitable.*
☐	☒	For Bayesian analysis, information on the choice of priors and Markov chain Monte Carlo settings
☒	☐	For hierarchical and complex designs, identification of the appropriate level for tests and full reporting of outcomes
☐	☒	Estimates of effect sizes (e.g. Cohen's *d*, Pearson's *r*), indicating how they were calculated

Our web collection on statistics for biologists contains articles on many of the points above.

Software and code

Policy information about availability of computer code

Data collection	No software was used for data collection
Data analysis	Parasite genomics:<br>Mapping of sequence reads were done using SMALT v.0.7.4<br>Genome wide variant calling (SNPs, INDELs) was done using GATK v.4.0.1.0<br>The final sets of SNPs and INDELs were annotated with SNPEFF v4.5<br>The frequencies of the alternate allele read depths were calculated using the vcf2freq.py script (available at github.com/FreBio/mytools).<br>NeighborNet networks were reconstructed with SplitsTree v.4.17.0<br>The population genomic structure was examined with ADMIXTURE v.1.3.0 and fineSTRUCTURE v.4.1.1.<br>Individual genotypes were phased with BEAGLE v.5.2<br>Local ancestry was assigned with PCAdmix<br>F3-statistics were calculated with Treemix v1.13<br>Decay of LD was calculated and visualized using PopLDdecay R package v2.7.5<br>Environmental Niche Modeling (ENM) was done using Maxent v.3.4.3, as implemented in the ‘dismo’ R package v1.3.14<br>Key analyses scripts and input data for the landscape genomic analyses are available from https://github.com/sheerenbiol/LandGenLeish.<br><br>Viral genomics:<br>Raw RNA sequencing reads were trimmed and filtered with fastp v0.20.1 and assembled de novo with MEGAHIT v1.2.9<br>Assemblies were improved with Pilon v.1.23, examined with Artemis v18.1.0 and aligned with MAFFT v.7.49<br>Maximum likelihood trees were generated using IQ-TREE v.1.6.12<br>Pairwise genetic distances were calculated with the ‘ape’ R-package v5.7

Nucleotide diversity and Fst statistics were calculated using the 'PopGenome' R package v2.7.5
Tests for recombination were done in SplitsTree v4.15.1
The Shannon diversity index was calculated using the 'vegan' R package v2.6.2
Phylogeographic analyses were done using BEAST v.1.10.4 and the BEAGLE library v.4.0.0.
Co-phylogenetic analyses were done using the phytools R package v1.0.3

Statistical analyses were done using the 'stats' R package v4.0.5 (Fisher's exact test, chi-squared test, Kruskal-Wallis test), the 'fmsb' R package v0.7.5 (pairwise Fisher's exact test), the 'elrm' R package v1.2.5 (exact logistic regression) and the 'FSA' R package v0.9.4 (pairwise Dunn's test).
R v4.0.5 and Rstudio v2023.03.1 were used for statistical and downstream analyses.

For manuscripts utilizing custom algorithms or software that are central to the research but not yet described in published literature, software must be made available to editors and reviewers. We strongly encourage code deposition in a community repository (e.g. GitHub). See the Nature Portfolio guidelines for submitting code & software for further information.

Data

Policy information about availability of data

All manuscripts must include a data availability statement. This statement should provide the following information, where applicable:

- Accession codes, unique identifiers, or web links for publicly available datasets
- A description of any restrictions on data availability
- For clinical datasets or third party data, please ensure that the statement adheres to our policy

The metadata used for this study was the collection site for the 79 L. braziliensis isolates, which is given for each sample in Supplementary Table 1 (provided as Source Data file). Genomic sequence reads of the 79 sequenced L. braziliensis genomes are available in the European Nucleotide Archive (https://www.ebi.ac.uk/ena/browser/home) under accession number PRJEB4442 [https://www.ncbi.nlm.nih.gov/bioproject/PRJEB4442/]. The assembled sequences of the 31 LRV1 genomes are available in GenBank (https://www.ncbi.nlm.nih.gov/genbank/) under accession numbers OQ673070-OQ673100 [https://www.ncbi.nlm.nih.gov/nuccore/
OQ673070,OQ673071,OQ673072,OQ673073,OQ673074,OQ673075,OQ673076,OQ673077,OQ673078,OQ673079,OQ673080,OQ673081,OQ673082,OQ673083,OQ673084,OQ673085,OQ673086,OQ673087,OQ673088,OQ673089,OQ673090,OQ673091,OQ673092,OQ673093,OQ673094,OQ673095,OQ673096,OQ673097,OQ673098,OQ673099,OQ673100]. Source data are provided with this paper

Research involving human participants, their data, or biological material

Policy information about studies with human participants or human data. See also policy information about sex, gender (identity/presentation), and sexual orientation and race, ethnicity and racism.

Reporting on sex and gender	N/A
Reporting on race, ethnicity, or other socially relevant groupings	N/A
Population characteristics	N/A
Recruitment	N/A
Ethics oversight	N/A

Note that full information on the approval of the study protocol must also be provided in the manuscript.

Field-specific reporting

Please select the one below that is the best fit for your research. If you are not sure, read the appropriate sections before making your selection.

☐ Life sciences ☐ Behavioural & social sciences ☒ Ecological, evolutionary & environmental sciences

For a reference copy of the document with all sections, see nature.com/documents/nr-reporting-summary-flat.pdf

Ecological, evolutionary & environmental sciences study design

All studies must disclose on these points even when the disclosure is negative.

Study description	Study involves the use of cryo-preserved parasites to investigate the population diversity and structure of Leishmania braziliensis and Leishmaniavirus 1 in Peru and Bolivia. Quantitative data was not obtained as part of this study.
Research sample	All parasites were collected during previous studies on the genetics and epidemiology of leishmaniasis in the region. At the time, parasite isolates were obtained from cutaneous and muco-cutaneous leishmaniasis patients who presented at health facilities in Peru and Bolivia for care. These parasites were cryo-preserved at the Antwerp Institute of Tropical Medicine (ITM). Data collection during these previous studies was authorized by the ethical committees of Peruvian (Instituto de Medicina Tropical Alexander von

Humboldt, Lima) and Bolivian (Centro Universitario de Medicina Tropical, Cochabamba) partners, and approved by the Institutional Review Board of ITM.
Within the context of this study, we decided to grow all cryo-preserved parasites on a blood medium and extracted their DNA/RNA at ITM. Sequencing was subsequently done at the Wellcome Sanger Institute.

Sampling strategy	The sampling procedure at the time involved obtaining samples from leishmaniasis patients, and the number of samples depended on the number of patients presenting at health facilities in Peru and Bolivia. There was no predetermined sample size: the rationale was to capture as much as possible the diversity of Leishmania parasites and Leishmaniaviruses in the region.
Data collection	The collection of parasites at the time were done by our partners in Peru and Bolivia (including Jorge Arevalo, Lineth Garcia, Alejandro Llanos-Cuentas) and trained nurses. The recording of the sample ID, sampling location and time was done by the field staff and trained nurses.<br>Cryo-preserved parasites were grown in vitro on blood medium in order to obtain nucleic acids for sequencing, which was done by I. Maes at ITM. Sequencing was done by M. Sanders at Sanger Institute.
Timing and spatial scale	Samples were obtained between 1984 and 2003 from 25 different localities in Peru and Bolivia. These samples were collected during multiple studies investigating the genetics and epidemiology of leishmaniasis in the region. The exact location and timing of sampling, together with the latitude and longitude, can be found in Supplementary Table 1, as described in the paper.
Data exclusions	All data were included for sequencing. Three parasite genomes were subsequently removed for downstream analyses because they showed aberrant allele read depth frequencies, as described in the paper.
Reproducibility	Regarding the experimental data, we evaluated the correctness of viral genome assemblies by comparing them to partial viral sequences. Details of the concordance between viral assemblies and partial sequences are described in the paper.<br>All other results were based on describing the parasite en viral genome diversity using a set of parasite isolates that were collected during previous studies. Concordance in results (in particular parasite and viral population structure) as obtained from different analyses tools reflect the reproducibility of our study. Results can be reproduced by following the detailed methods section.
Randomization	Randomization was not relevant to the study, as all cryo-preserved parasites were subjected to sequencing in order to capture as much as possible the full extent of parasite and viral diversity. Hence, randomization was not applicable.
Blinding	The Investigators were not blinded to allocation during experiments and outcome assessment. Blinding was not relevant to the study.

Did the study involve field work? ☐ Yes ☒ No

Reporting for specific materials, systems and methods

We require information from authors about some types of materials, experimental systems and methods used in many studies. Here, indicate whether each material, system or method listed is relevant to your study. If you are not sure if a list item applies to your research, read the appropriate section before selecting a response.

Materials & experimental systems

n/a	Involved in the study	
☒	☐	Antibodies
☒	☐	Eukaryotic cell lines
☒	☐	Palaeontology and archaeology
☒	☐	Animals and other organisms
☒	☐	Clinical data
☒	☐	Dual use research of concern
☒	☐	Plants

Methods

n/a	Involved in the study	
☒	☐	ChIP-seq
☒	☐	Flow cytometry
☒	☐	MRI-based neuroimaging

Plants

Seed stocks	N/A
Novel plant genotypes	N/A
Authentication	N/A
